# A Case Report of Human Herpesvirus-6 (HHV-6) Meningitis Masquerading as Idiopathic Intracranial Hypertension in an Immunocompetent Patient

**DOI:** 10.7759/cureus.4636

**Published:** 2019-05-10

**Authors:** Hayden Z Smith, Richard Paguia, John Horne, Manasa Velagapudi

**Affiliations:** 1 Infectious Diseases, Creighton University Medical Center, Omaha, USA

**Keywords:** headache, viral, hhv-6, roseola, immunocompetent, aseptic meningitis, pseudotumor cerebri, idiopathic intracranial hypertension, nucleic acid amplification testing

## Abstract

We present a case of a 32-year-old woman with signs and symptoms of idiopathic intracranial hypertension (IIH), but who, upon further investigation, was found to have human herpesvirus-6 (HHV-6) in both cerebrospinal fluid (CSF) and serum. This rare cause of meningitis in an immunocompetent individual and a relatively unique presentation is described along with a review of proper diagnostic workup and treatment. HHV-6 meningitis is commonly detected via molecular diagnostics and thus needs confirmatory testing of viral load of acellular compartments or viral serology. The reason for this added diagnostic step is due to the incorporation of the virus into the host DNA, leading to increased false-positive results on screening tests. In this case, proper diagnosis, treatment, and follow-up were pursued by following guidelines proposed in the literature of HHV-6 meningitis.

## Introduction

The majority of adults and children have been exposed to and retain seropositivity against the ubiquitous human herpesvirus-6 (HHV-6) [1]. After primary infection, HHV-6 can incorporate into the host genome and remain dormant in peripheral blood mononuclear cells, neural cells, and other brain tissues [2-4]. This can lead to a life-long latent infection that may reactivate during times of immunosuppression or stress.

In patients with meningitis of unknown etiology, HHV-6 has been shown to be present in up to 40% of cerebrospinal fluid (CSF) isolates [3]. This makes the primary infection or viral reactivation of HHV-6 an emerging explanation for central nervous system (CNS) infections where no clear pathogen can be isolated. For CNS infections involving immunocompromised patients, guidelines are clear on how to diagnose, manage, and follow the patient. But, guidelines are not as well researched on proper workup of immunocompetent patients with meningitis of unknown origin [5-7]. This problem is compounded when uncommon pathogens are suspected and mimickers like IIH must be ruled out [8].

We report this case of confirmed HHV-6 meningitis with symptoms that resembled IIH in an immunocompetent patient. We primarily outline a presentation of HHV-6 due to presumed reactivation that is not previously reported in the literature [4,9]. Additionally, we suggest workup, treatment, and diagnosis for this example and discuss the reasons behind that action.

## Case presentation

History and physical

A previously healthy 32-year-old Caucasian woman with a past medical history significant for obesity (body mass index (BMI) 48.42), depression, and asthma presented to the emergency department (ED) with a daily headache for five weeks. She reported no headaches at baseline. Starting just over a month prior to the presentation, the patient began to have headaches that occurred two or more times a week. The frequency increased dramatically and on her initial ED visit, she was sent home on naproxen. A week later, she went to the ED again, reporting that her headache continued to occur multiple times a day at varying times, lasted several hours, and had variable foci. The headache was associated with photophobia, blurred vision, nausea, and vomiting. It was refractory to oral (PO) pain medications and sumatriptan. She denied any aura or flashes of light. The review of systems was positive for occasional chills and negative for fever, cough, wheeze, and runny nose.

Patient history was significant only for recent exposure to a respiratory syncytial virus through her daughter. She denied any recent travel and lived with two dogs at home. The patient reported occasional alcohol use and being a current smoker of one to two cigarettes per day. Family history was noncontributory.

On examination, she was afebrile, normotensive, and bradycardic, with a heart rate of 51/min and a respiratory rate of 18/min. She was oriented to person, place, and time; her pupils were round, equal and reactive; and she was found to have bilateral papilledema on physical exam. She had no focal deficits. The rest of the physical examination was normal. Therapeutic and diagnostic lumbar puncture (LP) was performed with opening pressure (OP) 38 cmH_2_O, and the patient reported an improvement in headache following LP.

Investigation

**Figure 1:**
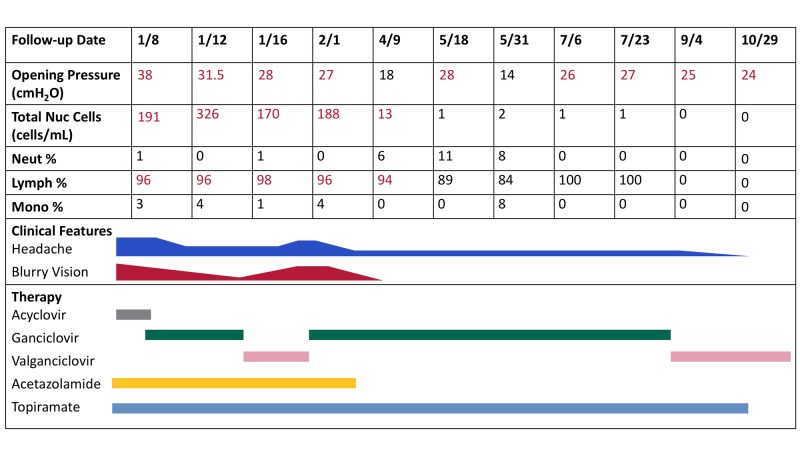
CSF Labs with Clinical Course The patient's clinical course is reflected by the intensity of symptom bars, namely, headache and blurry vision. These are juxtaposed with LP opening pressures, white cell counts, and therapy given at the time of assessment. ​​​​​​ CSF: cerebrospinal fluid; LP: lumbar puncture

Initial workup included a normal magnetic resonance imaging (MRI) head venogram and CSF analysis demonstrating a lymphocytic pleocytosis with increased protein levels and normal glucose concentration (Figure 1). Gram stain of CSF was negative for any organisms. Subsequent testing on CSF included meningitis/encephalitis nucleic acid amplification panel, which indicated the presence of HHV-6. Herpesvirus-6 immunoglobulin M (IgM) was 1:20 (negative as per interpretation) and herpesvirus-6 IgG resulted as 1:40. Basic labs did not indicate any acute infectious process and human immunodeficiency virus (HIV) panel showed negative HIV serology. After admission, other causes of aseptic meningitis were investigated and all tests came back negative, including Cryptococcus, Histoplasma, Coccidioides, and Mycobacterium. Subsequently, the patient’s initial CSF and serum were sent out for HHV-6A and HHV-6B viral load due to negative workup for aseptic meningitis. The results of testing showed an extremely high viral load at >999,999 viral copies/mL but were delayed three months due to outsourcing.

Differential diagnosis

While awaiting confirmatory test results, many differential diagnoses for a central nervous system (CNS) infection of unknown origin were entertained. Initial admission to hospital was for IIH, but other potential causes included: viral meningitis (enterovirus, herpes simplex virus (HSV), HHV, West Nile, human immunodeficiency virus (HIV)), fungal meningitis (Cryptococcus, Histoplasma, and Coccidiodes), Lyme disease, intracranial arteriovenous malformations, status migrainosus, and intracranial hemorrhage.

Treatments

In the ED, the patient was given prophylactic acyclovir 10 mg/kg q8h and acetazolamide 500 mg bid for potential HSV infection and presumed IIH. Eventually, meningitis/encephalitis nucleic acid amplification testing (NAAT) detected HHV-6 DNA in the CSF and acyclovir was changed to ganciclovir 2.5 mg/kg twice daily despite no confirmatory testing proving HHV-6 as the cause of the patient’s meningitis [10-12]. Upon admission for increased intracranial pressures, both acetazolamide and ganciclovir were continued for symptom relief, IIH coverage, and possible HHV-6 infection. Four days after the first LP, a second LP showed persistently elevated opening pressure but, this time, caused a worsening headache. Neurology was consulted and the patient was started on topiramate 25 mg qhs. On hospital day 16, the patient’s headaches improved to four episodes per day and she was discharged to home on acetazolamide, topiramate, and PO valganciclovir 900 mg bid [13].

Outcomes and follow-up

Three weeks post-discharge, the patient was seen by her infectious disease provider and was found to have worsening symptoms. She complained of increasing headache, nausea, vomiting, visual disturbances, and neck stiffness. There was recurrent papilledema on examination, and she was admitted for worsening viral meningitis. LP was performed, which showed an elevated opening pressure of 27 cmH20, and CSF continued to be positive for HHV-6 DNA on NAAT. The patient was transitioned back to original ganciclovir dose and symptoms improved. She was discharged four days later on intravenous (IV) ganciclovir.

**Figure 2:**
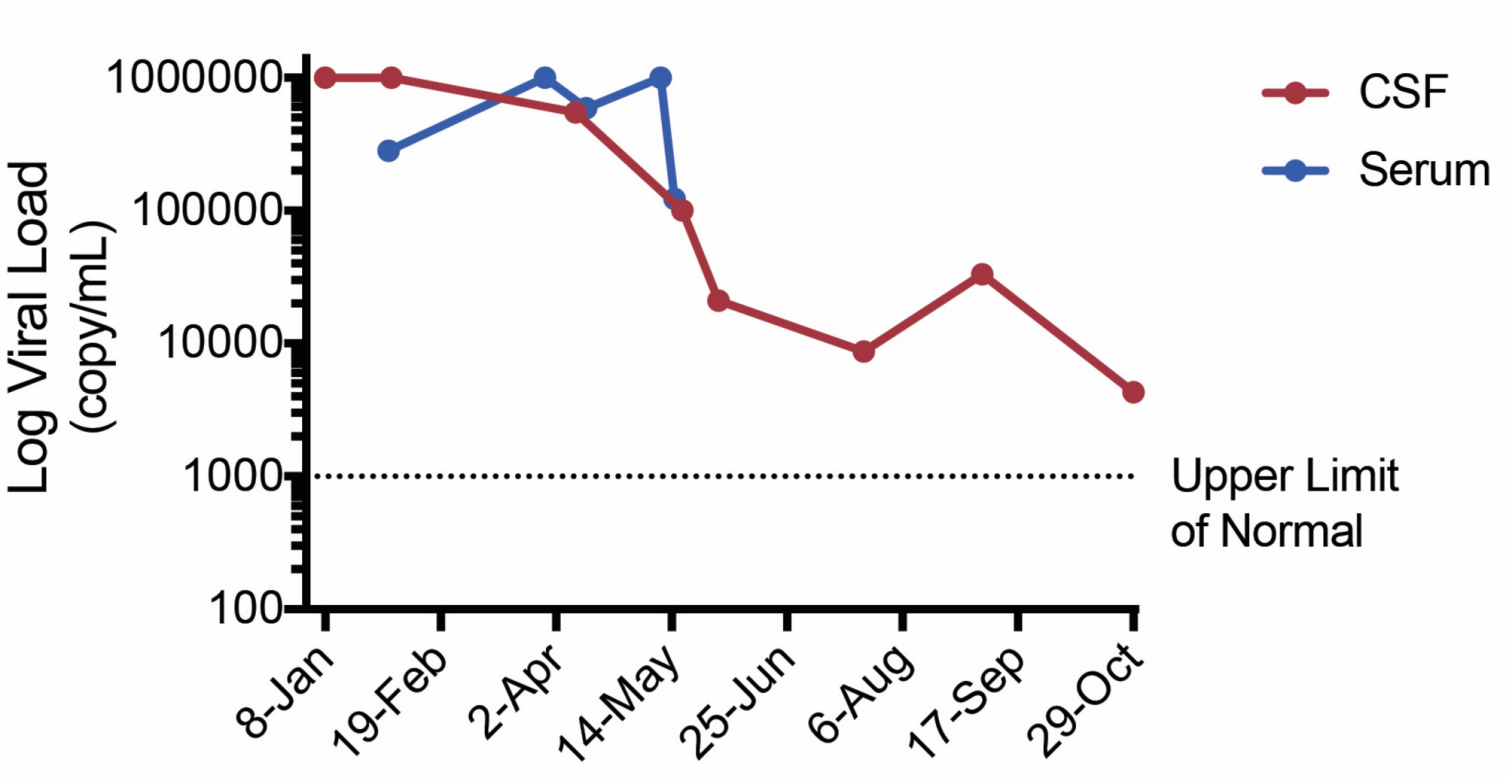
HHV-6 Viral Load Over Time Patient CSF and serum viral load were assessed simultaneously and represented on a viral load log scale. Long-term viral load was tracked via CSF and correlated with clinical presentation. The upper limit of normal for viral load testing was defined as 1000 viral copies per mL. CSF: cerebrospinal fluid; HHV-6: human herpesvirus-6

The outpatient course has since been stable, with mild to moderate headache occurring multiple times a week. Follow-up serial LP normalized with OP 18-27 cmH2O and IIH medications were stopped. HHV-6 viral load was periodically assessed and has decreased to as low as 4,290 viral copies/mL (Figure 2). She was switched to PO valganciclovir therapy after nine months of intravenous (IV) ganciclovir and continues to have longer headache-free periods and is tolerating therapy well. The plan is to continue antivirals with the goal of a viral load <1,000 viral copies/mL.

## Discussion

This case represents a more benign disease course when compared to other instances of HHV-6 meningitis in the literature. Even in immunocompetent individuals, previous reports illustrate a clinical picture that consisted of hallucinations, parathymia, apraxia, and aphasia [11]. Other cases demonstrated fever, quadriparesis, and generalized hyperreflexia, or more classic meningeal symptoms with fever, headache, confusion, irritability, and drowsiness [9,12,14]. A review of 31 cases of HHV-6 meningitis in immunocompetent adults revealed a spectrum of disease presentation can be seen and there appears to be no consistent presentation of HHV-6 meningitis [1-2,4,7,9-15]. The reasons behind such variable signs and symptoms between patients are unknown. Diverse patterns of neuroinvasion and reactivation of HHV-6 may play a part [2].

Due to the rarity and diverse disease presentation of HHV-6 meningitis in the immunocompetent population, there is no standardized workup and treatment. In the cases reviewed, the most common diagnostic step is a NAAT of CSF, blood, or tissue, and it was done *de novo* or as a part of a viral meningitis panel. This may not be the most accurate way to diagnose HHV-6 [12]. One study by Yao et al. proposes how to properly run NAAT screening and recommends confirmatory testing after NAAT is required for definitive diagnosis [3-4]. As a herpesvirus, HHV-6 has the ability to incorporate into the host genome by remaining latent in peripheral blood mononuclear cells, neural cells, and other brain tissues after a primary infection, usually from childhood. It is from this latent state that the virus reactivates as an adult to cause the symptoms previously discussed and seen in this case. Yao continues to emphasize that it is for this reason all NAAT testing should be done in an acellular compartment like CSF or serum plasma due to the ability of NAAT testing to detect latent viral DNA in host cells. The frequency of HHV-6 DNA detection in peripheral blood lymphocytes and saliva range from 5%-98% in healthy patients and up to 40% in the CSF of patients with encephalitis symptoms of unknown origin [3,14]. A multicenter evaluation of the BioFire FilmArray (BioFire Diagnostics, Utah, US) meningitis/encephalitis assay reported that HHV-6 has the worst sensitivity of all pathogens tested at 85.7% and a specificity of 99.7%. When the discrepancy of false positivity is investigated though, the rate of false positivity for HHV-6 increased to 75% [16]. Thus, once screening with NAAT indicates that HHV-6 is positive, further confirmation of disease is required [3-4,7]. Either serology or the viral load of acellular fluids has been proposed as confirmation of HHV-6 meningitis [3,11].

After a definitive diagnosis, the treatment for HHV-6 meningitis entails a backbone of either ganciclovir or valganciclovir therapy [9,12]. The consensus in the literature appears to be that ganciclovir should be used in more virulent disease whereas valganciclovir is used when symptoms are well-controlled. However, as this report demonstrates, the patient may or may not tolerate one antiviral over the other and clinical judgment is advised in those situations. The duration of therapy is even more variable from case to case. Due to its quantifiable nature, it's proposed viral load could be used to track disease progression and therapeutic duration as was done in this case until standardized treatment options become available since no concordant timelines were found in the literature [1-2,4,7,9-15].

## Conclusions

The prevalence of HHV-6 can be a complex question and one that must be answered in the context of its propensity to incorporate into host DNA. The major pitfall to such a diagnosis is the ease by which NAAT testing extracts HHV-6 DNA from blood and CSF to hair follicles and tissue. This diagnostic problem, coupled with incongruent reports of HHV-6 meningitis in immunocompetent patients, suggests that one must have due diligence in diagnosis. Thus, extensive workup for HHV-6 meningitis after routine screening should be postponed until after other avenues have been considered due to a high likelihood of false positivity in NAAT. Here is a case where initial diagnostic tests of aseptic meningitis of unknown origin were inconclusive but screening showed HHV-6 positivity. Once more, the common etiologies of aseptic meningitis were effectively ruled out, and a definitive workup for HHV-6 meningitis was pursued and confirmed.
